# Remimazolam and Remifentanil Use Induced Severe Respiratory Depression and Laryngeal Spasm During Intravenous Sedation and Analgesia: A Case Report

**DOI:** 10.2174/1574886318666230517101142

**Published:** 2023-11-10

**Authors:** Zhijun Xin, Ning Wang, Huaizhou Wang

**Affiliations:** 1 Yantai Stomatological Hospital, Yantai, Shandong Province, 264000, China

**Keywords:** Intravenous sedation, analgesia, remifentanil, remimazolam, respiratory depression, laryngospasm

## Abstract

**Introduction:**

Intravenous sedation and analgesia are widely used in minor surgeries. Remifentanil and remimazolam are advantageous in this setting because of their rapid onset of action, and short duration of action leading to a rapid recovery. However, the two drugs combined need to be titrated to avoid airway-related adverse events.

**Case Presentation:**

This article reports a case of severe respiratory depression and severe laryngeal spasm induced by remifentanil and remimazolam when they were used for analgesia and sedation in a patient undergoing oral biopsy.

**Conclusion:**

We aim to improve awareness about the safety of these drugs among anesthesiologists and increase their ability to manage the risk associated with their use.

## 1 INTRODUCTION

Laryngospasm is a common complication in clinical anesthesia. The factors that induce it are aspiration, airway hyper-responsiveness, airway stimulation, *etc*. [1]. Mild and moderate laryngospasms are more common in the perioperative period and can often be relieved by irritant removal, oxygen inhalation, mask ventilation, and intravenous medication. Severe laryngospasm is a serious complication that may be life-threatening if not treated promptly [2]. We have, herein, reported a case of severe respiratory depression and severe laryngeal spasm in a patient undergoing oral biopsy where remifentanil and remimazolam were used for analgesia and sedation. We intervened in a timely and effective way and saved the patient’s life. Based on this case, we hope that clinical anesthesiologists will pay more attention to the safety of these drugs to prevent adverse outcomes.

## 2 CASE PRESENTATION

A 66-year-old woman, with a body mass index (BMI) of 28.9 and a history of type 2 diabetes for 10 years, received daily subcutaneous protamine and human insulin mixed injection at 16 U 15 minutes before breakfast and dinner, and her fasting blood glucose was controlled at 7 to 8 mmol/L. She also had a history of multiple myeloma for 1 year. She was on once weekly subcutaneous bortezomib 3.5 mg, daily oral dexamethasone acetate, and daily oral cyclophosphamide. The patient's blood and urine workups, coagulation function, ECG, and chest X-ray were normal. The blood biochemistry was as follows: blood glucose: 8.05 mmol/L (normal value 3.33-6.11 mmol/L), uric acid: 446.91 umol/L (142-340 umol/L), lactate dehydrogenase: 391.26 U/L (135-214 U/L), total cholesterol: 7.15 mg/L (0.0-5.67.15 mg/L), and creatinine: 106.62 umol/L (44-80 umol/L). The admission diagnosis was as follows: 1). right maxillary mass, 2). dentition defect, 3). type 2 diabetes, 4). multiple myeloma, 5). hypercholesterolemia, and 6. hyperuricemia. We planned to perform an oral biopsy for the maxillary mass under intravenous compound anesthesia.

After the patient was admitted to the operating room, we established intravenous access and cardiac monitoring, and then administered oxygen by a double-port nasal cannula at 5 L/min. The patient’s blood pressure was 143/85 mmHg, heart rate was 75 beats/min, SPO_2_ was 97%, and respiratory rate was 19 breaths/min. The anesthesiologist used a 0.9% saline solution to dilute remimazolam and remifentanil. The ratio of remimazolam was 36 mL:36 mg, and the ratio of remifentanil was 50 mL:1 mg. The prepared drugs were drawn with a 50 mL syringe and placed into an intravenous infusion pump. The two drugs were pumped separately through two infusion channels in the pump. The initial pumping dose was set at 0.1 mg/kg/h for remimazolam and 0.05 µg/kg/min for remifentanil. After 5 minutes, the anesthesiologist observed that the patient had no discomfort, was conscious, and could answer questions. The surgeon used 1.5 mL of articaine with adrenaline (1:100,000) for the superior posterior alveolar nerve block. After the patient complained of pain, the anesthesiologist used an intravenous pump to quickly inject remifentanil 1 mL (0.02mg). After about 3 minutes, the anesthesiologist did not receive a reply after communicating with the patient. The ECG monitor showed the SPO_2_ to be decreased to 93%, and it did not significantly improve after the nasal catheter oxygen flow was adjusted to 8 L/min. When the SPO_2_ decreased to 86%, the anesthesiologist suspected that the patient had respiratory depression and immediately used pressurized mask ventilation. The patient's mask ventilation pressure and respiratory resistance were very high. Meanwhile, SPO_2_ decreased to 22%, heart rate increased to 108 beats/min, and ECG showed atrial premature contraction. The SPO_2_ was maintained at 23% to 60% without significant improvement. The anesthesiologist suspected the patient to be experiencing laryngeal spasm or bronchospasm and they immediately administered intravenous methylprednisolone 40 mg, propofol 100 mg, fentanyl 0.15 mg, and mivacurium 12 mg before using a visual laryngoscope for endotracheal intubation. During intubation, the glottis was closed, which caused great resistance. After pulling out the catheter core, the tracheal catheter slipped out of the glottis. The second intubation was successfully inserted using a 7.0 tracheal tube. There was no abnormality found during lung auscultation. The P_ET_CO_2_ values were 55 mmHg. After mechanical ventilation, SPO_2_ gradually improved and was maintained at 97% to 99%, and the P_ET_CO_2_ gradually returned to normal and was fixed at 40 to 45 mmHg. The surgery was continued under general anesthesia, and was successful. After surgery, the patient was fully awake and able to answer questions fluently. Her Steward score was 6, and she was returned safely to the ward.

## 3 DISCUSSION

An oral tissue biopsy is an oral and maxillofacial surgery that lasts a short time and the use of local infiltration or nerve-blocking anesthesia is usually sufficient. However, in many cases, patient comorbidities may affect the safety of surgery. Intraoperative monitoring by anesthesiologists is often required, and some sedative and analgesic drugs can be given to reduce nervousness and pain depending on the type of surgery and the patient's condition. Anesthesiologists often choose intravenous sedation and analgesia either as single agents or in combination to reduce surgical irritation and make the patient comfortable. Both drugs are safe and effective [3, 4]. Drugs with a rapid onset of action, short duration of maintenance, rapid recovery, and few adverse reactions are usually used. Remimazolam is a new ultra-short-acting and water-soluble benzodiazepine with the same sedative effect and anterograde amnesia as midazolam [5], but a more rapid onset of effect, shorter maintenance time, and recovery time [6]. Also, it is suitable for target-controlled infusion (TCI), has no injection pain compared to propofol, and has a lower incidence of hypotension and respiratory depression [7]. Presently, it is mostly used for induction and maintenance of sedation or general anesthesia in short surgeries and examinations. Remifentanil is an ultra-short-acting analgesic commonly used in clinical anesthesia that has similar advantages of rapid onset, short elimination half-life, no accumulation, and rapid postoperative recovery.

Administration of remifentanil and sedative drugs provides a better anesthetic effect [8]. Early data suggest that remimazolam and remifentanil combined can induce and maintain anesthesia. A study has shown that this combination used in painless gastroscopy maintained stable vital signs and enhanced the safety of anesthesia [9]. Another study showed the use of the combination in general anesthesia for ureteroscopic lithotripsy in the elderly to have a good anesthetic effect, induce stable hemodynamics, and significantly improve postoperative cognitive function. However, the combination tended to induce respiratory depression and even apnea [10]. The Guideline for Prescribing Opioids for Chronic Pain issued by the Centers for Disease Control and Prevention suggests that opioids should not be taken with benzodiazepines to avoid respiratory depression [11]. A review showed that opioids combined with benzodiazepines increase the risk for severe adverse respiratory events and mortality over a broad range of clinical and non-clinical settings [12]. Midazolam combined with fentanyl is a popular technique for achieving moderate sedation, but unexpected hypoxemia is frequently reported [13]. Therefore, anesthesiologists should be attentive to the adverse effects of this combination.

Adverse reactions from remimazolam are rare in clinical studies. The most common ones listed in the prescribing information are hypotension, heart rate reduction, and respiratory depression. A study by Hu *et al.* [14] on 346 elderly patients who underwent gastroscopy compared remimazolam (0.2 mg/kg) with propofol (1.5 mg/kg). There was a lower incidence of respiratory depression and other sedative-related adverse events found in the remimazolam arm [14]. Chae *et al.* [15] successfully estimated the ED50/ED95s of intravenous bolus remimazolam from the time to loss of consciousness and respiratory depression. They proposed optimal remimazolam doses of 0.25 to 0.33 mg/kg, 0.19 to 0.25 mg/kg, and 0.14 to 0.19 mg/kg in patients aged younger than 40, 60 to 80, and older than 80, respectively. These were based on the ED95 estimates for the corresponding age groups. The maximum effect of remimazolam is achieved within 3 minutes, and it is unaffected by body weight (the 65-90 kg group was studied). The peak sedation effect of remimazolam was observed within 1 to 2 minutes at doses greater than 0.075 mg/kg [16, 17]. An animal test involving cynomolgus monkeys showed remimazolam as a potent sedative drug and to exhibit a fast onset, being dose-dependent, and showing a high degree of synergism with remifentanil [18]. Compared to fentanyl, remifentanil has a rapid onset and offset of action, a stable half-life, and it can provide similar analgesic and respiratory depressant effects [19]. Babenco *et al.* [20] suggested that respiratory depression occurs as early as 2.5 minutes after a single intravenous infusion (0.5 µ/kg) of remifentanil. However, Bouillon *et al.* [21] emphasized, in their study, that remifentanil is a potent analgesic and respiratory depressant with an effective concentration (EC50) of 0.92 ng/mL for ventilatory depression. Therefore, a clinically significant hypoventilation state, such as a slowed respiratory rate or a rise in PETCO_2,_ is most likely to occur especially with a single intravenous push.

The research by Lobb *et al.* [22] suggested that when remifentanil concentration was fixed at 1.0 ng/mL or less, a TCI of remifentanil combined with midazolam (5.021 ± 1.507 mg) could shorten the recovery time and reduce respiratory changes without increasing adverse effects in patients undergoing dental procedures under moderate procedural sedation. There was no overdose of remimazolam or remifentanil in our case. Therefore, the severe respiratory depression and laryngeal spasm could be because of the following reasons: 1). Respiratory depression induced by combined remimazolam and remifentanil, especially after a single injection of remifentanil aggravated the course of respiratory depression and subsequent airway adverse events. 2). Our patient had a history of multiple myeloma controlled with bortezomib, cyclophosphamide tablets, and dexamethasone tablets, which are immunosuppressive drugs that could lower immunity [23]. Also, a history of type 2 diabetes, which is often combined with peripheral nerve or autonomic nervous system pathology, could easily cause reduced neural reflexes and cognitive function [24, 25]. Multiple studies have shown that diabetic patients who have delayed gastric emptying are considered as having a full stomach because they are at an elevated risk of aspiration [26, 27]. The fasting guidelines of the American Society of Anesthesiologists (ASA) for elective surgeries do not consider patients with certain co-morbidities, such as diabetes [28]. Almost half of the patients with type 2 diabetes are considered to have a full stomach based on current preoperative fasting guidelines [26]. Consequently, patients might be more likely to have a reflux of stomach contents that leads to aspiration and laryngeal spasms. 3). The glottic protective reflex induced by oral secretions or aspiration causes the glottis to close and interfere with effective ventilation. This combined with severe respiratory depression caused by remifentanil and remimazolam may have led to the onset of laryngeal spasm and severe persistent hypoxemia in our case.

Generally, anesthesiologists confidently handle the respiratory depression caused by intraoperative sedative and analgesic drugs. Transient respiratory depression can be solved by awakening the patient to deepen and increase their breathing rate. Respiratory depression combined with a loss of consciousness usually indicates a high sedative or analgesic dose. Oxygen inhalation or mask ventilation can relieve serious respiratory depression. Patients with respiratory depression combined with laryngeal spasms are in a relatively critical situation, and emergency airway event management procedures need to be initiated [26]. Laryngospasm is divided into mild, moderate, and severe. Mild laryngeal spasms can be relieved spontaneously after removing the inducing factors. Moderate laryngospasms mostly require a facemask-pressurized oxygen supply. Severe laryngeal spasms may result in complete occlusion of the glottis and airway. This requires emergency intra-airway intervention to establish effective ventilation or intravenous administration of the short-acting muscle relaxant succinylcholine to relieve spasms while oxygen is given under a facemask. If it still cannot be relieved, immediate tracheal intubation is required for artificial or mechanical ventilation [27, 28]. It has been shown that in severe laryngospasm, inward and forward pressure in the area between the mandibular angle and the mastoid process (the pressing position is shown in Figs. (**1** and **2**) combined with a jaw-holding maneuver known as the “Larson maneuver” can effectively stimulate deep breathing and restore voluntary ventilation and oxygenation in patients. In another study, the success rate of laryngeal spasm relief was significantly improved when facemask-pressurized oxygen was combined with moderate chest compressions (the assistant pressed the sternum with the root of the palm of their hand at about half the depth of standard CPR chest compressions 20-25 times/min). In addition to the above measures, intravenous corticosteroids (*e.g*., dexamethasone, hydrocortisone, *etc*.) or propofol can have a good effect [29-33].

## CONCLUSION

Our anesthesiologist managed this patient with severe respiratory depression and laryngospasm in a timely and effective way, but this case also revealed some concerns. First, they failed to carefully consider the patient's illness and medication history, and thus did not anticipate the possible intraoperative anesthetic risks. Second, the dose of anesthetic medication was not titrated from small doses, and the risk of a single injection of remifentanil was not considered. Finally, the PETCO_2_ was not monitored under existing conditions to detect respiratory depression or apnea in the first place. Therefore, anesthesiologists need to pay attention to potential problems, adopt a risk awareness attitude of “only small surgery, no small anesthesia”, strictly assess patients preoperatively, carefully use medication, and strictly monitor patients intraoperatively to limit such situations from occurring.

## Figures and Tables

**Fig. (1):**
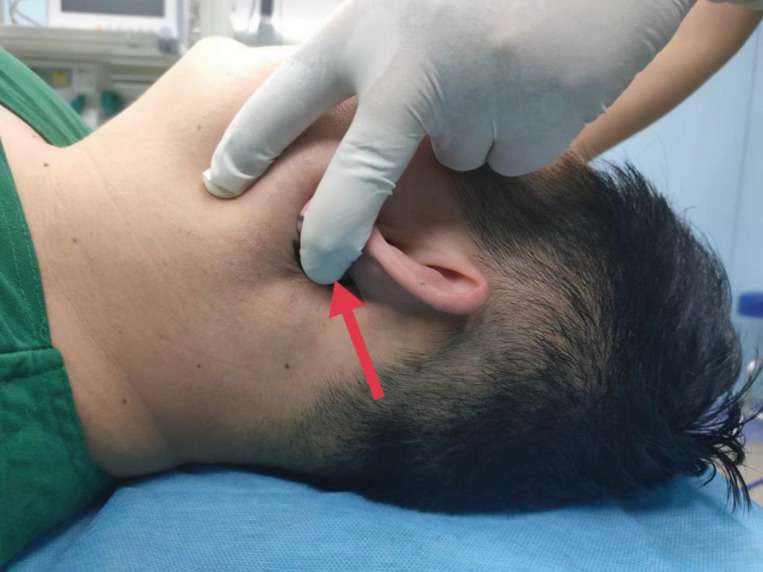
The red arrow indicates the pressing position of Larson's maneuver.

**Fig. (2):**
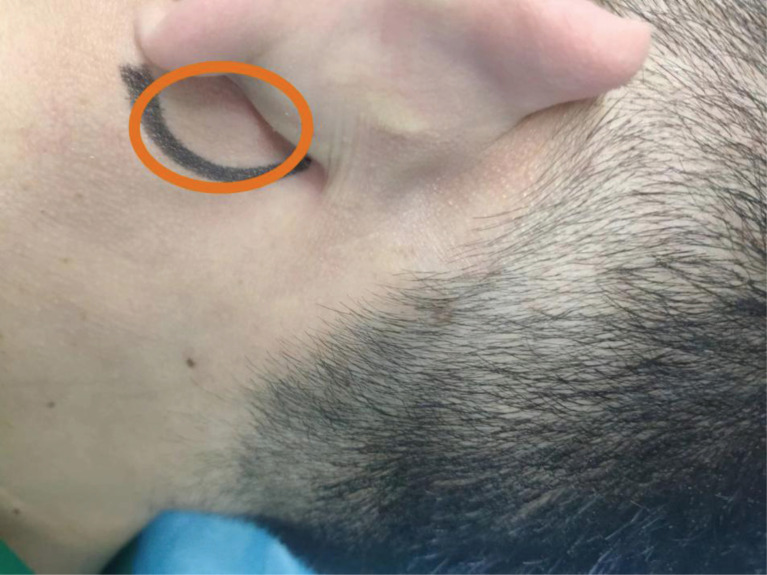
The elliptical portion indicates the range of Larson's maneuver, which is bounded by the mandibular condyle in the front, the mastoid in the rear, and the skull base in the upper part.

## Data Availability

All the data and supportive information are provided within the article.

## LIST OF ABBREVIATIONS

BMI: Body Mass Index
ASA: American Society of Anesthesiologists
TCI: Target-Controlled Infusion
